# Capturing Intrusive Re-experiencing in Trauma Survivors’ Daily Lives Using Ecological Momentary Assessment

**DOI:** 10.1037/a0034957

**Published:** 2013-11

**Authors:** Birgit Kleim, Belinda Graham, Richard A. Bryant, Anke Ehlers

**Affiliations:** 1 Institute of Psychiatry, King’s College London, London, United Kingdom, and University of Zurich, Switzerland; 2 Institute of Psychiatry, King’s College London, London, United Kingdom; 3 University of New South Wales, Sydney, Australia; 4 Institute of Psychiatry, King’s College London, London, United Kingdom and University of Oxford, Oxford, United Kingdom

**Keywords:** PTSD, intrusion, trauma, diary, ecological momentary assessment

## Abstract

Intrusive memories are common following traumatic events and among the hallmark symptoms of posttraumatic stress disorder (PTSD). Most studies assess summarized accounts of intrusions retrospectively. We used an ecological momentary approach and index intrusive memories in trauma survivors with and without PTSD using electronic diaries. Forty-six trauma survivors completed daily diaries for 7 consecutive days recording a total of 294 intrusions. Participants with PTSD experienced only marginally more intrusions than those without PTSD, but experienced them with more “here and now quality,” and responded with more fear, helplessness, anger, and shame than those without PTSD. Most frequent intrusion triggers were stimuli that were perceptually similar to stimuli from the trauma. Individuals with PTSD experienced diary-prompted voluntary trauma memories with the same sense of nowness and vividness as involuntary intrusive trauma memories. The findings contribute to a better understanding of everyday experiences of intrusive reexperiencing in trauma survivors with PTSD and offer clinical treatment implications.

Spontaneous and emotion-laden intrusion of traumatic memories is a core feature of posttraumatic stress disorder (PTSD), one of the most frequent psychological problems following trauma (American Psychiatric Association, 1994). Intrusions comprise mostly sensory impressions from moments of the trauma, such as visual, auditory, or bodily sensations, and emotional responses from the trauma (Ehlers, Hackmann, & Michael, 2004). They tend to occur “out of the blue,” and survivors are often unaware of what triggered the memory: a motor vehicle accident (MVA) survivor may suddenly reexperience the sight of headlights coming toward him, just like before the accident; an assault survivor may see the assailant’s face in front of him, just like during the assault. The intrusiveness of these memories, along with a “here- and-now” quality, contributes to a sense of current threat, as the sensory memories from the trauma may be experienced, without realization that they are from a past event (Ehlers & Clark, 2000; Ehlers et al., 2002).

Intrusive memories are not unique to PTSD and are common in the initial months after trauma. This raises the question of whether there are features of intrusive trauma memories that are predictive of PTSD. Evidence shows that there may be few differences between trauma survivors with and without PTSD in presence and frequency of intrusions (Michael, Ehlers, Halligan, & Clark, 2005). However, a sense that the memory content is happening in the present, as well as a lack of context of the intrusion and the distress associated with it, were strong predictors of concurrent and later PTSD. Another large-scale study found that whereas intrusive phenomena were common across disorders after trauma, only flashback memories that involved a sense of reliving the experience were distinctive of PTSD (Bryant, O’Donnell, Creamer, McFarlane, & Silove, 2011). There may also be differences between trauma survivors with and without PTSD in their emotional response to intrusive trauma memories. In addition to fear, emotional responses such as anger and humiliation, guilt or shame (Andrews, Brewin, Rose, & Kirk, 2000; Brewin, Andrews, & Rose, 2000; Ehlers, Mayou, & Bryant, 1998; Grey, Holmes, & Brewin, 2001), and sadness (Berntsen & Rubin, 2007; Dalgleish & Power, 2004) have all been shown to be elevated in trauma survivors with PTSD. The emergence of these emotions in direct response to intrusive memories has so far only been investigated retrospectively. One study retrospectively indexed a range of emotions experienced in the context of intrusive memories in patients with PTSD and found anger and sadness to be higher in response to an intrusion than at the time of the trauma, while this was not the case for anxiety and helplessness (Speckens, Ehlers, Ruths, & Clark, 2007). In a second study, patients with depression and PTSD identified anger, sadness, fear, helplessness, and guilt as emotions that most frequently accompany intrusions (Reynolds & Brewin, 1999).

The individual’s behavioral response to an intrusive trauma memory may play a key role in the development and maintenance of PTSD symptoms following a traumatic event. Trauma survivors use a range of strategies in response to the experience of intrusive memories. One study found that specifically those with PTSD tend to interpret intrusions as signs of impending threat, hence inferring danger from the presence of intrusions (Engelhard, Macklin, McNally, van den Hout, & Arntz, 2001). According to Ehlers and Steil (Ehlers & Steil, 1995), individuals who assign their intrusive memories a threatening meaning are more likely to find their intrusions distressing and to engage in avoidance strategies such as rumination, dissociation and suppression, thus increasing the probability that intrusive memories and PTSD are maintained. In line with this hypothesis, negative interpretations of PTSD symptoms predicted PTSD, both concurrently and prospectively (Clohessy & Ehlers, 1999; Dunmore, Clark, & Ehlers, 2001; Ehlers et al., 1998; Steil & Ehlers, 2000). Similarly, rumination, suppression, as well as dissociation in response to intrusions have been shown to maintain PTSD (Ehring, Ehlers, & Glucksman, 2008; Laposa & Rector, 2012; Murray, Ehlers, & Mayou, 2002). Such cognitive–behavioral strategies have in common that they prevent conceptual processing of the trauma memory and changes in appraisals of the trauma, and may thus lead to further occurrence of intrusive memories and maintenance of PTSD (Ehlers & Clark, 2000).

A key characteristic of intrusive memories is that they are retrieved involuntarily. Whether and how they differ from voluntarily recalled trauma memories, and whether this difference may be specific to PTSD, is less clear. Theory and clinical evidence suggests that involuntary intrusive trauma memories are experienced as perceptual in PTSD (Ehlers et al., 2002; Michael, Ehlers, Halligan, & Clark, 2005), and that they tend to be accompanied by decrements in voluntary conceptual trauma memory retrieval (Brewin, Gregory, Lipton, & Burgess, 2010). Voluntary recall of the series of events experienced during the trauma may be disjointed and poorly organized in PTSD, with details missing and difficulties recalling the exact temporal order of events and other relevant autobiographical information (Halligan et al., 2003; Ehlers & Clark, 2000; Kleim, Wallott, & Ehlers, 2007). There is also evidence that trauma survivors tend to recall the moments of the trauma that are reexperienced in a more disorganized way than other moments from the trauma and may even omit them from their trauma narratives (Evans et al., 2007).

Different theoretical models of involuntary trauma memories have been put forward, with conflicting views regarding how such memories are represented in memory. One view is represented by a single representational system account positing that trauma memories use the same representation as other autobiographical memories and differ only in retrieval processes thought to be associative and bottom-up rather than strategic and top down. These processes may thus lead to some of the differences found in individuals with versus without PTSD (Berntsen, Rubin, & Bohni, 2008; Rubin, Berntsen & Bohni, 2008). Results from two recent diary studies are in line with this theory, in that they did not find PTSD-specific differences between voluntary and involuntary memories. The first study compared involuntary with voluntary memories in undergraduates and found that PTSD symptoms were associated with more emotionally negative and trauma-related memories, as well as more reactions and mood change (Rubin, Boals, & Berntsen, 2008). Involuntary and voluntary recall followed the same pattern of results, regardless of PTSD symptoms. A diary study of community dwelling adults replicated these results in participants with and without PTSD diagnosis including the finding that PTSD was associated to a tendency to react with intense emotional responses to *all* memories, irrespective of whether they were involuntary or voluntary memories (Rubin, Dennis, & Beckham, 2011).

Another view is represented by theories assuming multiple memory systems as responsible for voluntary and involuntary memories. Dual representation theory (DRT; Brewin et al., 2010), for instance, posits two types of memory representations, consisting of flexible, abstract, and contextually bound representations (C-reps) that become integrated into personal semantic memory over time and inflexible, lower level sensory representations (S-reps). It is assumed that, in healthy individuals, S-reps for emotional events become connected to corresponding C-reps, hence allowing integration in appropriate semantic and autobiographical contexts and increasing top-down control of the representation. Images formed in the respective neural sites along with C-reps may nevertheless activate S-reps normally contributing to the sensory aspects of the memory. In PTSD, dysfunctional encoding is thought to lead to relatively stronger S-reps, weaker C-reps and an impaired connection between the two. S-Reps may therefore be created without this association to corresponding C-reps, hence resulting in intrusive reexperiencing. A number of studies found differences for voluntary and involuntary trauma memory recall in PTSD, when comparing intrusive flashbacks of the trauma and ordinary trauma memories, and thus support DRT. Intrusive flashbacks differed from voluntarily recalled memories in being more detailed and more likely to involve emotions such as fear, helplessness and horror (Hellawell & Brewin, 2004) and were associated with increased autonomic and motor behavior (Hellawell & Brewin, 2002). Note, however, that these comparisons were about different moments from the trauma. It is therefore unclear whether the differences were because of retrieval mode or because of different encoding or memory characteristics of different parts of the trauma. Given (a) that only a few moments from the trauma are reexperienced, whereas much of the trauma is not (Hackmann et al., 2004), and (b) Evans et al.’s (2007) findings that memory characteristics and the ease of voluntary retrieval differs between the worst moments (which are represented in reexperiencing) and other moments from of the trauma, it appears important to compare voluntary and involuntary memories of the *same* moments in memory. The present study therefore expands on the previous studies and directly compares involuntary intrusive and voluntary memories with the same content in trauma survivors with and without PTSD by prompting individuals to recall the content of their main intrusive memories voluntarily. Because some of the earlier studies were limited by the nature of the samples that often did not include a clinical PTSD diagnosis, the present study included clinically diagnosed trauma survivors.

The studies reviewed so far mostly indexed summarized accounts of intrusive memory characteristics retrospectively, via interviews or questionnaires. Whereas initial studies have investigated involuntary and voluntary memories in real life of nonclinical populations, such as undergraduates (Ball & Little, 2006; Berntsen & Rasmussen, 2011; Brewin, Christodoulides, & Hutchinson, 1996; Kvavilashvili & Mander, 2004; Mace, 2004; Rubin & Berntsen, 2009), surprisingly little is known about intrusive trauma memories as they occur in trauma survivors’ everyday lives. While a handful of small-scale diary studies have investigated intrusive trauma memories and their impact on mood (Berntsen, 2001; Rubin, Dennis, & Beckham, 2011), no study has examined intrusive reexperiencing of trauma memories along with distinct emotional and behavioral responses as they occur, in trauma survivors’ natural environment.

## A Daily Diary Approach to Studying Intrusions

Retrospective summary reports of “typical” experiences of intrusive reexperiencing require participants to recall, average, and summarize their experiences and are subject to considerable error, because disproportionate weight may be given to highly significant past instances relative to current or ongoing events (Myin-Germeys, Oorschot, Collip, & Lataster, 2009). They are thus limited by recall biases and do not capture symptom or behavior changes across time and contexts (Sato & Kawahara, 2011).

Ecological momentary assessment (EMA) involves repeated sampling of actual momentary experiences in subjects’ naturalistic environment and in real time (Shiffman, Stone, & Hufford, 2008), which makes it less vulnerable to such recall bias. It has been used to assess stress, depression, schizophrenia and other psychopathology symptoms (Myin-Germeys et al., 2009). EMA thus captures data within people’s natural lives, rather than in the artificial surroundings of a laboratory, hence improving ecological validity. Multiple assessments may be associated with each entry, including assessments of context and reactions. An additional feature of EMA is that participants can be prompted at random intervals by the diary device to respond to specific questions. Given these features, EMA appears particularly suited to assess intrusive memories in trauma survivors’ everyday lives, because it allows for a study of microprocesses surrounding intrusive reexperiencing, such as the immediate emotional and cognitive–behavioral response, as well as potential stimuli that triggered an intrusion. The possibility to prompt individuals to voluntarily recall trauma memories enables a direct comparison of characteristics of voluntary memories with involuntary intrusive trauma memories.

## The Current Investigation

We indexed intrusive memories in trauma survivors with and without PTSD using electronic diaries allowing for a detailed and accurate assessment and understanding of intrusion characteristics, including intrusion triggers, and specific emotional responses to intrusions close to when they occur. The present study also included a comparison of matched involuntary and voluntary memories of the trauma. A better understanding of intrusive memories has implication for theoretical accounts of PTSD, for understanding underlying mechanisms, as well as treatment implications.

Aims of the current study were fourfold. First, we aimed to examine the characteristics of intrusive memories in trauma survivors’ everyday lives using EMA. Results of prior studies reviewed above led to the prediction that those with PTSD would experience their intrusions as more vivid and with a greater sense of nowness than those without PTSD. We had no hypothesis regarding group differences in frequency. Second, we aimed to investigate whether emotional responses differ between individuals with and without PTSD, and posited that those with PTSD would experience stronger direct emotional responses to their intrusions. Third, we aimed to explore within-person associations between intrusion characteristics, emotional and cognitive–behavioral responses; that is, the joint fluctuation of the variables under study within each person across memories. Finally, we investigated whether involuntary intrusive memories differed from voluntary memories of the same moments during the trauma. We prompted participants at random times throughout the week to voluntarily recall the content of their most frequent intrusive trauma memory. DRT (Brewin, 2011) suggested the hypothesis that in PTSD intrusive involuntary memories would mainly be supported by S-reps and therefore be experienced as more vivid and with a greater sense of newness than voluntarily recalled memories.

## Method

### Participants

Assault and motor vehicle accident (MVA) survivors were recruited through flyers and local advertisements. Inclusion criteria, assessed over the phone, included (a) experienced an assault or MVA that met the trauma A1 criterion specified in *Diagnostic and Statistical Manual of Mental Disorders, Fourth Edition* (*DSM–IV*; American Psychiatric Association, 1994) and experienced at least one intrusive memory in the past week, (b) mastery of written and spoken English to complete assessment and questionnaires, (c) a minimum age of 18 years. Participants with current psychosis and substance dependence, as well as those who could not remember the event (e.g., because of a head injury) were excluded. Sixty-one of 96 individuals interviewed on the phone met these inclusion criteria and were invited to a research session. Fifty-two participants attended, 49 of whom completed the intrusion diary. Three of these participants were either noncompliant or completed the diary on the last two days only. Data will thus be reported for 46 participants. Trauma exposure ranged between 1.5 months and 44 years prior to the study (*M* = 4.7 years). Demographic and clinical characteristics are displayed in Table 1.[Table-anchor tbl1]

### Materials

A computerized questionnaire was developed to assess intrusive memories in trauma survivors’ everyday life. Participants identified their three most frequent intrusions relating to the index trauma in the initial session and this information was included in the personalized diary. For example, one RTA survivor identified the following three memories: memory 1 (most frequent intrusion): “Bus crashing into her, seeing it coming into her lane and hitting her,” memory 2: “Thinking she was dead, trying to get out of car,” memory 3: “Hearing woman screaming outside.” Some participants experienced less than 3 intrusions and provided these memories. Participants received instructions to record every intrusive trauma memory relating to the index trauma that they had throughout their waking day for the coming 7 days and to indicate which of the three identified intrusions they experienced, or whether it was an intrusion with a different content. Entries were, however, restricted to 1 entry per hour, to keep the recording burden reasonable for those experiencing multiple intrusive memories throughout the day. Participants were instructed to carry the electronic diary with them at all times, and to enter information on each intrusive memory as it occurred (event-based design).

Each intrusion entry was preceded by the question of whether the entry was a trauma memory. If participants indicated that this was not the case, they were thanked and the entry stopped automatically. For each trauma memory, participants were asked about the content of the intrusive memory, about intrusion characteristics (intrusiveness, vividness, nowness, each scored on a scale from 0 to 100), emotional response (anger, fear, guilt, shame, helplessness, each scored on a scale from 0 to 100), and cognitive–behavioral responses (dwelling, suppression, distraction, alcohol/medication intake, each scored as to whether or not participants had responded in this way following the intrusion). Participants were also asked whether they could identify a trigger for the memory, and asked to enter this information manually into the diary via the Palm’s keyboard. This information was scored according to 7 trigger types (Table 2) by a graduate level clinical psychologist experienced in PTSD; interrater reliability was high, κ = .91 (based on 30 intrusions, 2 independent raters). A total of 14 items were completed for each intrusion entered into the diary (see Appendix A for an overview of diary content).[Table-anchor tbl2]

Participants were also prompted to retrieve their most frequently occurring intrusive memory at random times 10 times throughout the week. They had 5 min to respond to each prompt. Participants responded to 2 prompts on average, *SD* = 2.19 (range = 0–9) and 10 participants did not respond to any prompt; those with and without PTSD did not differ in the number of responses to prompts, *F*(1, 44) = 1.45, *p* = .235. Prompted memories were compared with matched involuntary memories, that is, only of their most frequently occurring memory that they were also asked to retrieve in response to the prompt.

Palm Zire 22 handheld computers were used as recording devices. Diary questions were programmed and displayed using Satellite Form Software (Thacker Network Technologies Inc, Alberta, Canada). Responses were timestamped by the program. When returning the Palm computer, participants retrospectively answered questions regarding the representativeness of the diary week as well as possible reactive effects.

### Procedure

Participants attended a laboratory session where they provided informed consent, identified their three most common intrusive memories, completed some additional unrelated questionnaires and a computer task and received written and verbal instructions on how to use the Palm diary. They also completed a full practice trial. Each participant received a small handbook for personal use that explained the procedures and provided contact details in case of problems with the diary use. A second appointment was arranged to return the completed Palm diary. During this second appointment, participants were also interviewed with the CAPS to establish PTSD symptoms and diagnosis. Participants also completed the intrusion interview during the first session and some additional questionnaires about reactivity effects and frequency of intrusions in the week of keeping the diary during this second session. The protocol was approved by the local ethics review board. Participants received a reimbursement of $78 (£50) for participating in the study.

### Additional Self-Report Questionniare

The Beck Depression Inventory, BDI (Beck & Steer, 1987) is a widely used, standardized, and normed measure of severity of depression. The BDI asks participants to decide between four different response choices reflecting different degrees of symptom severity. Items were then scored from 0 to 3, with the sum of the item scores representing the total BDI score, ranging between 0 and 63. Internal consistency in the present study was very good (α = .93).

### Interviews

#### PTSD diagnosis

PTSD diagnosis was established with a standard structured clinical interview, the Clinician-administered PTSD Scale (Blake et al., 1995). The interviewer, a trained graduate psychologist, rated each of the PTSD symptoms for frequency and for intensity, each on a scale from 0 to 4. PTSD was rated as present if the participant reported the number of symptoms specified in *DSM–IV*. Interrater reliability was high, κ = .80 (based on 10 interviews, 2 raters who were each uninformed as to the other rater’s diagnoses). A CAPS severity score was calculated by summing intensity and frequency ratings across all symptoms (range = 0–136).

#### Intrusion interview

Intrusion characteristics were assessed with an intrusion interview adapted from Hackmann et al. (2004). This interview identifies whether trauma survivors have intrusive memories, as well as the content and characteristics of these memories, that is, how often participants experienced the intrusive memory in the past week, whether the intrusion comprises one of the worst moments of the trauma, and from what point in time the intrusive memory is. In addition, modalities of intrusions are identified, as well as frequency, duration, and specific qualities of intrusions. For the current analyses, we used information on intrusion content for the personalized diaries. Participants were also asked in the second session, after returning the palm, for a retrospective account of how many intrusions they had experienced during the past week, that is, the week of keeping the diary.

### Statistical Analysis

Analyses were conducted with SPSS 19.0. We controlled for differences in time since trauma and, in the analyses of prompted memories, we controlled for percentage time of carrying the Palm along to account for differences in number of perceived prompts. Standard measures of effect size, that is, Cohen’s *d,* are reported for differences between PTSD and non-PTSD groups.

Because of the nested data structure, we used multilevel modeling (Mplus 5.0, Muthen & Muthen, 2003) for examination of associations between daily diary measures within individuals, that is, vividness and nowness ratings, emotions, and cognitive–behavioral strategies. In our study, multiple daily observations on intrusive memories (Level 1) were nested within participants (Level 2). Multilevel modeling simultaneously estimates within- and between-person effects (Krull & MacKinnon, 2001) while handling varying time intervals between entries and missing data (Bosker & Snijders, 1999), and does not assume independence of data points.

## Results

### Palm Diary Compliance and Comparison With Retrospective Intrusion Interview

Participants reported to have carried the Palm pilot with them at 84% of their time (*SD* = 18.8), without disruption to their normal week for the majority of participants (57%). Most participants (87%) reported that the number of intrusions experienced was in the average or higher than average range compared with a usual week. Participants with and without PTSD did not differ in these reports, all *p* < .177. Participants with more total intrusions recorded also reported a greater number of intrusions that they did not record in the diary, *r* = .37, *p* = .018. The mean number of intrusions reported in the diary and the retrospective interview were *M*_Diary_ = 7.31 (*SD* = 7.97) and *M*_Interview_ = 8.05, (*SD* = 16.36).^1^

### Intrusive Memories in Trauma Survivors’ Everyday Lives

Participants recorded a total of 294 intrusions during the diary period (range = 0–41). Only two participants (4%) did not experience any intrusive memories within the respective week. Of the three intrusions described by participants in the initial session, they experienced on average two different intrusion contents (range = 0 to 4 memories), *M* = 2.10, *SD* = .96. Participants were not always aware of what had triggered the intrusion and provided information on perceived triggers for 61%, *SD* = 27.2, of their intrusions entered into the diary. We classified trigger descriptions into seven categories (Table 2) and found triggers that were perceptually similar to the trauma to be most frequent, *M* = 48%, *SD* = 38.7, followed by triggers relating to this study, such as seeing the Palm pilot or arranging an appointment for the next research session, *M* = 12%, *SD* = 21.1, and newspaper or TV reports, *M* = 10%, *SD* = 20.2. Helplessness was the most strongly reported emotional response to intrusions, *M* = 42.8, *SD* = 24.6, followed by fear, *M* = 39.5, *SD* = 25.6, anger, *M* = 36.2, *SD* = 27.9, shame, *M* = 20.4, *SD* = 26.2, and guilt *M* = 19.2, *SD* = 23.0. Emotional responses did not differ for the two trauma types (assault or MVA), all *p* > .288. Of four behavioral strategies assessed in response to intrusions, suppression was the most frequently reported behavioral response, *M* = 42.1%, *SD* = 29.6, followed by distraction, *M* = 34.0%, *SD* = 29.2, dwelling, *M* = 29.2%, *SD* = 25.9 and self medication with alcohol and/or drugs, *M* = 4.3%, *SD* = 10.6.

### Within-Person Associations Between Intrusion Characteristics, Emotional Response, and Cognitive–Behavioral Strategies

We calculated within-person associations between intrusion characteristics, that is, standardized slope estimates of the relation between Level 1 variables. These capture within-person associations between diary variables, across individual measurement occasions. As displayed in Table 3, significant relationships emerged between intrusion characteristics and emotions. Associations between emotions were significantly positive, with the exception of a significant negative association between fear and shame in response to intrusions. Perceived vividness of an intrusion was not significantly associated with emotional or behavioral responses. Perceived nowness, however, was significantly related to experiencing more fear, helplessness, as well as more intense guilt and shame. Neither vividness nor nowness were significantly associated with cognitive–behavioral responses. No associations between emotions and cognitive–behavioral strategies emerged.[Table-anchor tbl3]

### Intrusion Characteristics, Triggers, Responses and Association With PTSD

Participants with PTSD reported only marginally more intrusions than those without PTSD during the week of keeping the diary, *p* = .084, *d* = .51. These results are displayed in Table 4. The groups did not differ either in percentage of perceived intrusion triggers, 65.3%, *SD* = 28.3 (non-PTSD) versus 61.1%, *SD* = 20.2 (PTSD), *F*(1, 42) = .82, *p* = .371, *d* = .17, or in types and prevalence of intrusion triggers experienced, all *p* > .234. However, there were significant differences in intrusion quality and emotional responses to intrusions between participants with and without PTSD. Those with PTSD experienced their intrusions as significantly more intrusive, *d* = .74, and with significantly more “here and now” quality, *d* = .66 compared with those without PTSD. Participants with PTSD also reported stronger emotional responses compared with participants without PTSD, fear: *d* = .74, helplessness, *d* = .91, anger, *d* = .76, shame, *d* = .62. Levels of guilt were generally lower compared with the remaining emotions and the diagnostic groups differed only marginally in their reported guilt levels in response to their intrusions, *d* = .52. Those with PTSD reported more alcohol and drug use in response to their intrusions, *d* = .60, no significant differences emerged for dwelling, suppression and distraction.[Table-anchor tbl4]

PTSD symptom severity was significantly and positively associated with fear, *r* = .40, *p* = .009, helplessness, *r* = .52, *p* < .001, anger, *r* = .54, *p* < .001, shame *r =* .38, *p* = .012, and guilt, *r* = .32, *p* = .037. Only the self-medication score was significantly associated with PTSD symptom severity, *r* = .45, *p* = .002.

### Influence of Retrieval Type and PTSD Diagnosis on Intrusion Characteristics

**Figure 1 fig1:**
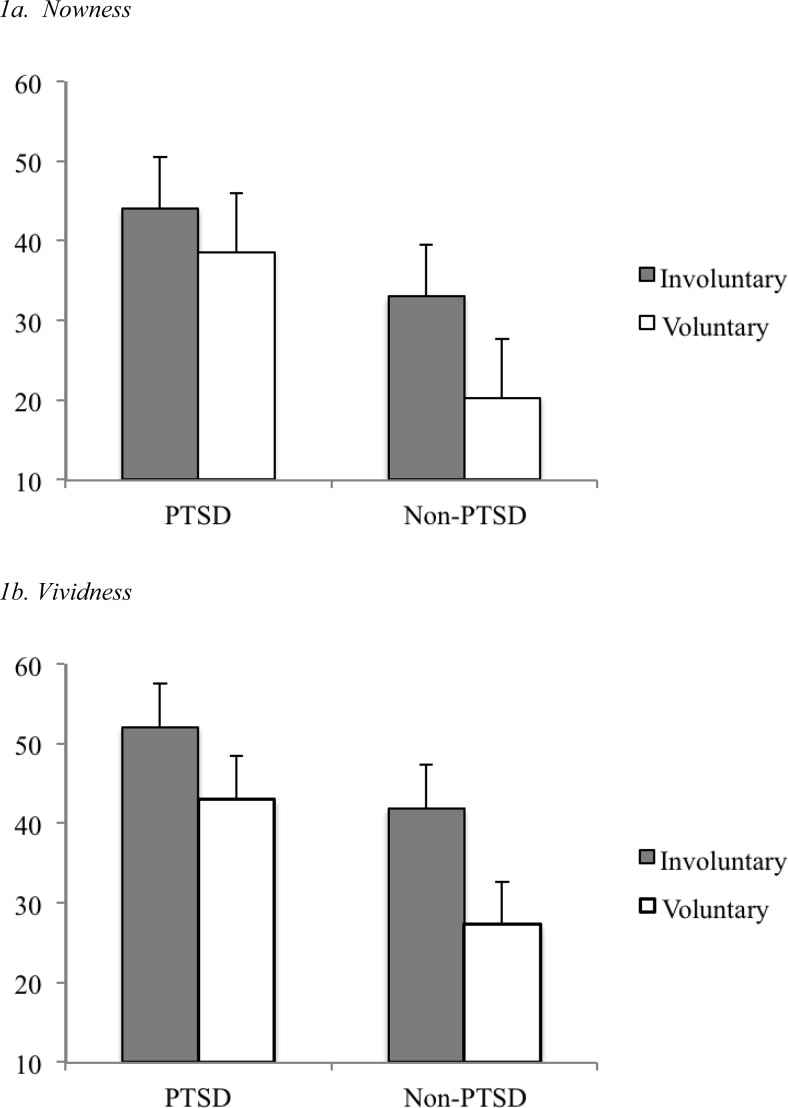
Differences in perceived nowness (1a) and vividness (1b) of involuntary versus voluntary trauma memories for posttraumatic stress disorder (PTSD) versus non-PTSD groups (*N* = 34). *Note.* Nowness and vividness were rated on a scale from 0 (*not at all*) to 100 (*extremely*); 10 participants did not respond to prompts. Error bars represent *SE*s.

A 2 (Retrieval mode: intentional vs. unintentional) × 2 (Diagnosis: PTSD vs. no PTSD) multivariate repeated measures analysis of covariance (MANCOVA) with the two covariates time since trauma and percentage of time of carrying the palm along was conducted with the dependent measures intrusiveness, vividness and nowness ratings.^2^ It revealed a significant effect of PTSD, *F*(1, 29) = 8.94, *p* = .006, indicating that those with PTSD experienced both involuntary and voluntary memories as more intrusive, vivid and with a greater sense of nowness than those without PTSD. There was also an effect of retrieval type, *F*(1, 29) = 8.11, *p* = .008, indicating that overall involuntary intrusive memories were perceived as more intrusive, vivid and with more sense of nowness than prompted voluntary memories. The critical interaction between retrieval mode and diagnosis was significant, *F*(1, 29) = 4.43, *p* = .044. Follow-up tests revealed that participants without PTSD experienced voluntarily retrieved memories as significantly less vivid, *t*(18) = 3.54, *p* = .002, and with less sense of nowness, *t*(18) = 2.97, *p* = .008 than involuntary intrusive memories (Figure 1). They showed a greater difference between voluntary and involuntary memories than those with PTSD, who failed to report significant differences in vividness and nowness, all *p* > .078. There was no significant difference for intrusiveness between the groups.[Fig-anchor fig1]

## Discussion

The present investigation captured intrusive reexperiencing of trauma memories in trauma survivors’ everyday lives using ecological momentary assessment. In particular, we examined intrusive trauma memory characteristics, triggers, as well as emotional, cognitive, and behavioral responses. Moreover, we investigated whether memory characteristics and responses to intrusions differ between trauma survivors with and without PTSD. Finally, we examined differences between involuntary intrusive memories and voluntarily recalled memories of the same moments during the trauma and whether involuntary trauma memories are experienced as particularly vivid and with a heightened sense of nowness in PTSD.

On average, participants recorded 7 intrusive trauma memories during the week of keeping the diary, with individual participants reporting up to 41 intrusions throughout the week. Some studies reported higher intrusion counts across similar time periods, such as Hackmann, Ehlers, Speckens, and Clark (2004) who found an average of 30 intrusions per week in participants diagnosed with PTSD about to undergo a course of cognitive behavior therapy, based on an intrusion interview (Hackmann et al., 2004). The present study included participants with low PTSD symptom levels, without PTSD diagnosis, who were not treatment-seeking. Moreover, our study likely underestimates trauma survivors’ intrusion frequency as we restricted intrusion entries to one intrusion per hour, to reduce participants’ recording burden. On average, participants reported intrusions of two different contents during the week. Intrusion content was thus mostly related to a small number of key moments from the trauma, which is congruent with studies on intrusive memories in various trauma survivor populations with and without PTSD (Hackmann et al., 2004).

Although participants in our study were not always aware of what had triggered an intrusion, the most frequently identified triggers were perceptual trauma reminders, such as seeing a car similar to the car involved in the accident, or hearing an ambulance siren. The fact that participants were unaware of a significant proportion of intrusion triggers is in line with associative learning theories of PTSD that posit that the associations formed at the time of the traumatic experience are pivotal in forming conditioned responses to these stimuli (Pitman, 1988). People with PTSD may preconsciously process information that is related to threat (Bryant & Harvey, 1995). These models accord with systematic observations of triggers of intrusive memories in PTSD that showed that the often comprise cues that do not have a strong meaningful relationship to the trauma, but that are temporally associated with the trauma and similar to the trauma in a particular modality, such as a pattern of light or a color present at the time of the trauma, which are often difficult for trauma survivors to spot (Ehlers et al., 2004). The finding is also in accord with results that healthy participants often retrieve emotionally positive memories involuntarily without awareness of triggers (Berntsen, 1998; Berntsen & Rubin, 2002). Our diary data thus largely corroborate earlier reports on the frequency and phenomenology of intrusions derived from questionnaire and interview studies on intrusive memories in trauma survivors.

However, we also expand on these studies as summarized accounts of intrusions are unable to capture within-person fluctuations of the association between intrusions and emotional responses. These studies mostly report either an association between peritraumatic emotions and intrusions (Evans, Ehlers, Mezey, & Clark, 2007) or general distress or fear associated with intrusive reexperiencing (Marshall, Schell, & Miles, 2010; Michael et al., 2005), as well as heightened anger and sadness in the context of reexperiencing. All of these studies used between-person assessments. The present daily diary study assessed intrusion-emotion associations within each person across multiple occasions, and we assessed several distinct emotional responses. As expected, within-person fluctuations in nowness of intrusions were related to concurrent fluctuations in the intensity of emotions. When intrusions were experienced with greater sense of nowness, participants reported a corresponding increase in fear, helplessness, guilt and shame. Intrusion characteristics and emotions were not significantly related to cognitive–behavioral responses. This may, however, be because of the categorical answer format of cognitive–behavioral strategy items in our study, which may have precluded sufficient response variance and therefore led to nonsignificant results. It is interesting that more shame in response to an intrusion concurred with less fear at the within-person level. These within-person measurements capture short-term relationships between shame and fear across intrusion occurrences. Additional analyses showed that, on the between-person measurement level, greater overall levels of shame were associated with greater levels of fear, *r* = .56, *p* < .001, indicating that participants who experienced more shame also experienced more fear. Within participants, however, the data suggest that if shame evolves in the context of an intrusion it is less likely that fear will be experienced at the same time. This is in accord with emotion theories asserting that different emotions tend to be associated with distinct appraisals (e.g., Siemer, Mauss, & Gross, 2007) and cognitive theories of PTSD (e.g., Ehlers & Clark, 2000). Shame is thought to result from negative appraisals about the self and one’s reactions during the trauma whereas fear is thought to result from appraisals about external threat. These two emotions may therefore be negatively associated in the context of different intrusions within individuals.

We examined differences between participants with and without PTSD with regards to intrusion characteristics and emotional responses to intrusions. While those with PTSD reported only marginally more intrusions and did not differ in their ability to spot intrusion triggers or in trigger characteristics, some important differences emerged. Participants with PTSD experienced their intrusions with a greater sense of nowness, replicating Michael et al.’s (2005) findings. Moreover, the intensity of most emotions in response to intrusions differentiated between trauma survivors with and without PTSD, and were positively associated with PTSD symptom severity, replicating Michael et al. (2005) and Rubin et al. (2011). Although some studies indicate that survivors of interpersonal trauma show more intense emotional reactions compared with accidental injuries or illnesses (Amstadter & Vernon, 2008), emotional reactions to intrusions did not differ depending on trauma type (assault or MVA) in our sample. Participants with PTSD experienced more fear, helplessness, anger, and shame in response to their intrusions than those without PTSD. This is in accord with a study by Hellawell and Brewin, who found more helplessness and fear associated with flashbacks in narratives of people with PTSD (Hellawell, 2002), as well as other studies proposing dominant emotions in PTSD other than fear (e.g., Andrews, Brewin, Rose & Kirk, 2000; Ehlers & Clark, 2000; Hathaway, Boals, & Banks, 2010; Holmes, Grey, & Young, 2005). Together, these results underscore the importance of a comprehensive coverage of different emotional consequences of trauma in PTSD diagnostic criteria and treatment. Emotions such as anger or shame tend to remain stable or increase after trauma (Amstadter & Vernon, 2008), and are thus important targets in psychotherapy for PTSD that may require therapeutic interventions in addition to or augmentation of techniques proven to be effective, such as exposure therapy (Harman & Lee, 2010; Lee, Scragg, & Turner, 2001).

We recorded whether participants employed four types of cognitive–behavioral strategies in response to intrusions, and found that they most often engaged in suppression of or distraction from an intrusive memory. Although self-medication was used only infrequently in the overall sample, that is, in response to less than 5% of intrusions, we found significant differences between the diagnostic groups. Those with PTSD engaged more often than the non-PTSD group in self-medication as a response to intrusive memories. Self-medication in response to intrusions may thus be one possible pathway contributing to the increased risk for substance use disorders in individuals with PTSD (Breslau, Davis, & Schultz, 2003). Participants with and without PTSD did not differ in the absolute frequency with which they employed the remaining strategies. We note that our categorical assessment format might not have captured nuanced differences in intensity between PTSD and non-PTSD groups. Moreover, the cognitive–behavioral questions in our diary were limited and we did not assess cognitive appraisals of intrusions, which have been shown to be associated with the development and maintenance of PTSD, and have been shown to motivate individual maladaptive coping strategies (Ehlers et al., 1998; Steil & Ehlers, 2000).

Are involuntary intrusive trauma memories different from memories of the same content that are prompted by the Palm with the instruction to retrieve them voluntarily? We expected that those with PTSD would experience both types of trauma memories as more vivid and with a heightened sense of nowness compared with those without PTSD, but that this effect would be particularly pronounced for intrusive, compared with voluntarily recalled trauma memories in PTSD. Our results show that, while those with PTSD indeed experienced trauma memories as more vivid and with a greater sense of nowness than those without PTSD, there was no significant difference between voluntary and involuntary memories in this group. When survivors with PTSD tried to voluntarily retrieve the moments of the trauma that they often retrieved involuntarily, they retrieved them with a comparable sense of nowness and vividness as the distressing involuntary intrusive memories they reported throughout the week. Participants without PTSD, on the other hand, showed a differentiation between voluntary and involuntary trauma memories. They experienced voluntarily retrieved trauma memories as significantly less vivid and with less sense of nowness than the involuntary intrusive memories they recorded. Ceiling effects can be ruled out as an explanation, as the ceiling for both vividness and nowness scores was not reached. Dual representation theory may offer one possible explanation for this finding (see Brewin et al., 2010). That is, participants were prompted by the diary to voluntarily retrieve the content of their most frequent intrusion. For most trauma survivors, this may have constituted the worst moment of the trauma, or a particularly difficult aspect of the trauma or its consequences. DRT suggests that in PTSD, these moments are predominantly laid down as S-reps, in a situationally accessible memory store. Access to these representations may operate particularly fast in PTSD, and at times bypass voluntary retrieval of C-reps and be retrieved by corresponding emotional states or environmental sensory cues. Trauma survivors with PTSD may thus experience a direct and fast access to S-reps that overrules voluntary retrieval and directly leads to the involuntary retrieval route. This may result in the lack of differences in vividness and nowness scores for both memory types in participants with PTSD. In other words, thinking back to the worst parts of the trauma and trying to retrieve these memories deliberately may be difficult for people with PTSD. This corresponds to Evans et al.’s (2007) finding that the moments that were reexperienced were often omitted from intentionally retrieved trauma narratives. The results are also compatible with Ehlers and Clark’s (2000) model of PTSD, which focuses on memory processes rather than representations. This model explains the “nowness” of memories as the result of poor integration of the worst moments of trauma (e.g., the moment when the person thought they were going to die) with other relevant information in memory that puts the meaning of the worst moments into perspective such as the knowledge that they have not died. Until this updating information is integrated and linked to the memory of this particular moment, the original meaning of the memory will be retrieved and it appears to happen in the here and now.

In contrast, participants without PTSD reported greater vividness and nowness of memories for involuntary and voluntary retrieval. The result for this group is in line with single-representation theories and with previous findings that involuntary trauma memories are experienced as more emotional than voluntary memories (e.g., Rubin, 2008). From the perspective of DRT, it is at first sight unexpected, but may reflect the fact that some individuals in this sample had subclinical levels of PTSD and integration of C-reps and S-reps may also have failed to occur in this group. They may, however, be better at avoiding unwanted activation of S-reps, hence leading to premature inhibition of processing (Brewin, Dalgleish, & Joseph, 1996).

The current study is not without limitations. The study was restricted to trauma survivors who experienced at least one intrusive memory per week. This may have underestimated the differences between the PTSD and no PTSD groups. The mean scores on the CAPS of the no PTSD group indicate subthreshold PTSD symptoms. Second, keeping the palm diary was demanding and time-consuming. Some participants did not record all intrusions and some did not respond to any of the prompts, although the majority indicated that they carried the palm with them for the majority of time. Participants entered intrusions in private, as they occurred during the course of everyday life. Compliance with the research protocol and completeness of entries and reactions to prompts are thus not guaranteed. Third, reactivity effects may have influenced intrusive reexperiencing in the week of keeping the diary, as some participants reported that the palm itself was a trigger for intrusive memories. However, the majority reported that reactivity effects were small. Fourth, the current approach did not distinguish between intrusive memories or flashbacks, which may be most characteristic of individuals with PTSD (Bryant et al., 2011), as well as between intrusive memories that were images versus those that were verbal thoughts, although this differentiation may be related to different mechanisms (Hagenaars, Brewin, van Minnen, Holmes, & Hoogduin, 2010). Other factors, such as context (Ehlers & Clark, 2000; Michael et al., 2005), trauma memory binding (Ehlers et al., 2004), or memory fragmentation (Brewin, 2011), may distinguish intrusive memories from PTSD versus non-PTSD and we did not assess these characteristics here. Fifth, although we instructed participants to index emotions and cognitive–behavioral strategies in response to intrusions, we cannot infer a causal direction. It is possible, for instance, that emotions may have led to or contributed to the development of an intrusive memory, rather than the intrusion leading to the emotional response. It is also likely that both processes influence each other and future studies could index these temporal processes around each intrusion using a more fine-grained diary assessment. Finally, we cannot compare intrusive trauma-related memories with other spontaneous memories of personal experiences as these were not assessed as part of this study.

Despite these limitations, the present findings have important implications. Our results allow for a more detailed understanding of the everyday experience of trauma memories in trauma survivors using a method with high ecological validity. They also contribute to a better understanding of how individuals with PTSD differ from trauma survivors without this disorder with respect to their intrusive trauma memory. Such information may usefully inform clinical practice. The finding that those with PTSD not only reported elevated levels of fear and helpessness, but also of anger and shame in direct response to their intrusions, for instance, points toward these emotions as an important target of PTSD therapy. In future studies, electronic diaries could be used during PTSD treatment to track and evaluate how intrusive memory characteristics change across treatment. EMA may provide important information on trauma memory triggers and may assist patients in learning to discriminate intrusive memories and their triggers from the actual traumatic experience. Using EMA, patients may be better able to discover and bring crucial information regarding their personal memories into treatment.

## Figures and Tables

**Table 1 tbl1:** Demographic and Clinical Characteristics (*N* = 46)

	PTSD (*n* = 20)	Non-PTSD (*n* = 26)	Difference test
Age (years), *M* (*SD*)	37.3 (18.1)	35.6 (13.3)	*F*(1, 45) = .13, *p* = .717
Education (years), *M*	14.2 (4.1)	14.8 (3.2)	*F*(1, 43) = .24, *p* = .625
Time since trauma (in months)	68.6 (102.8)	63.0 (101.3)	*F*(1, 44) = .033, *p* = .856
Female (*N*)	12	14	χ^2^(1) = .53, *p* = .376
Ethnicity			
Caucasian	7	16	χ^2^(1) = 14, *p* = .091
Non-Caucasian	12	10	
No information	1	0	
Employment			
Employed/studies	7	10	χ^2^(1) = .01, *p* = .912
Unemployed	12	16	
No information	1	0	
Verbal intelligence (NART), *M* (*SD*)	25.0 (9.5)	30.4 (7.5)	*F*(1, 44) = 4.57, *p* = .038
PTSD severity (CAPS), *M* (*SD*)	67.4 (10.4)	28.4 (14.0)	*F*(1, 44) = 104.91, *p* < .001
Depression severity (BDI), *M* (*SD*)	25.6 (10.1)	8.6 (7.7)	*F*(1, 44) = 41.0, *p* < .001
*Note*. PTSD = posttraumatic stress disorder; NART = National Adult Reading Test; CAPS = clinician administered PTSD Scale; BDI = Beck Depression Inventory.			

**Table 2 tbl2:** Percentages of Trigger Type Categories and Diagnostic Differences (*N* = 44)

Trigger type, % (*SD*)	Total sample (*n* = 44)	PTSD (*n* = 20)	Non-PTSD (*n* = 24)	Difference test, *p*
Perceptual, similar situation, stimulus or person	47.7 (38.7)	45.7 (35.2)	49.4 (42.0)	*F*(1, 43) = .10, *p* = .759
Physiological	6.9 (19.6)	9.2 (24.5)	5.0 (14.4)	*F*(1, 43) = .50, *p* = .482
Actual trauma scene	2.8 (11.8)	.4 (2.0)	4.7 (15.7)	*F*(1, 43) = 1.45, *p* = .235
Newspaper or TV reports	10.0 (20.2)	8.4 (14.2)	11.4 (24.4)	*F*(1, 43) = .24, *p* = .630
Trauma-related conversations	7.6 (14.7)	8.3 (14.8)	7.0 (15.0)	*F*(1, 43) = .10, *p* = .760
Trauma-related thoughts	4.1 (9.5)	2.4 (4.6)	5.6 (12.2)	*F*(1, 43) = 1.17, *p* = .286
Study-related cues	12.1 (21.1)	12.2 (18.7)	9.7 (21.2)	*F*(1, 43) = .65, *p* = .424
Others	8.9 (21.2)	10.6 (21.1)	7.4 (21.7)	*F*(1, 43) = .25, *p* = .619
No triggers perceived (*N*)	2 (4.5)	0	2 (8.3)	χ^2^ = 1.61, *p = .205*
*Note*. Two participants did not report any intrusive memories whilst keeping the diary.				

**Table 3 tbl3:** Within-Person Associations Between Daily Diary Intrusion Characteristics, Emotions and Cognitive-Behavioral Responses

	1	2	3	4	5	6	7	8	9
1. Vividness	—								
2. Nowness	.41	—							
3. Fear	.22	.45***	—						
4. Helplessness	.28	.54***	.66***	—					
5. Anger	.29	.40	.34	.45***	—				
6. Guilt	.03	.46***	.51***	.39	.22	—			
7. Shame	.21	.54***	−.37***	.78***	−.03	.49***	—		
8. Suppression	.11	.06	.12	.11	.14	−.03	.06	—	
9. Dwelling	.14	−.01	−.01	.02	.06	.02	−.05	−.49***	—
10. Distraction	.11	.10	.04	.06	.19	.01	.06	.19***	−.03
*Note*. Coefficients are based on standardized level 1 variables, Bonferroni correction was applied resulting in an adjusted p value of *p* < .001; *** *p* < .001; self-medication was not included in this analyses due to low count (4.3% of responses).									

**Table 4 tbl4:** Everyday Intrusion Characteristics, Emotional and Behavioral Responses to Intrusions by Participant Group (*N* = 44)

	PTSD (*n* = 20)	Non-PTSD (*n* = 24)	Difference test
Total number intrusions (*M*, *SD*)^a^	9.3 (9.8)	5.2 (5.7)	*F*(1, 45) = 3.11, *p* = .085
Frequencies^a^			
None	0	2	
1–5	9	16	
6–10	8	5	
>10	3	3	
Intrusiveness	34.1 (28.7)	17.2 (14.4)	*F*(1, 43) = 6.34, *p* = .016
Vividness	54.7 (26.9)	42.6 (21.0)	*F*(1, 43) = 2.82, *p* = .101
Nowness	45.9 (23.1)	30.0 (24.7)	*F*(1, 43) = 4.83, *p* = .033
Fear	49.5 (28.0)	31.3 (20.6)	*F*(1, 43) = 6.20, *p* = .017
Helplessness	54.0 (24.4)	33.4 (20.8)	*F*(1, 43) = 9.22, *p* = .004
Anger	47.3 (29.4)	27.0 (23.4)	*F*(1, 43) = 6.49, *p* = .015
Guilt	25.6 (26.6)	13.8 (18.3)	*F*(1, 43) = 3.0, *p* = .091
Shame	29.1 (30.2)	13.1 (20.22)	*F*(1, 43) = 4.39, *p* = .042
Dwelling	32.3 (23.9)	26.6 (27.7)	*F*(1, 43) = .52, *p* = .475
Suppression	42.6 (24.5)	41.7 (33.8)	*F*(1, 43) = .01, *p* = .920
Distraction	30.0 (20.2)	37.3 (35.1)	*F*(1, 43) = .69, *p* = .412
Self-medication (alcohol, drugs)	7.8 (14.2)	1.4 (4.6)	*F*(1, 43) = 4.41, *p* = .042
*Note*. Two participants did not report any intrusions during the diary period, mean intrusion characteristics and emotions range from 0 to 100; behavioral responses are reported in % employed in response to individual intrusions.			
^a^ Including 2 participants reporting no intrusions in the diary.			

## Footnotes

1 This is in accord with the instruction to not record more than one intrusion per hour.

2 Ten participants had not responded to the prompts, hence reducing the number of participants for this analysis.

## Intrusion Diary Content

Is the memory you are about to record a trauma memory? You do not need to record this memory. Only record memories related to the trauma 1. What was the memory about? NO YES Tick boxes (select one): memories identified in Session 1 + Other (with space for answer ________) 2. How much did you mean to think about this memory? Slider Control: 0 100 4. How much did you feel the memory was happening now rather than in the past? Slider Control: 0 100 6. To what extent did you feel each of these emotions in response to the memory? Slider Control: Anger: 1 100 Fear: 1 100 Guilt: 1 100 Shame: 1 100 Helplessness: 1 100 Please try to bring to mind your memory of... 7. What did you do when the memory popped into your head? Tick boxes (select multiple): Dwelled on it Tried to suppress it Distracted myself To o k a l c o h o l / drugs Other: ______ 5. What was the trigger for the memory? Space for short answer_______ Nb. Q only for intrusive memories Content Intrusiveness Vividness/ Nowness Trigger awareness Emotional response Response Strategies No response within 5 minutes
